# Correlation between CD4 counts of HIV patients and enteric protozoan in different seasons – An experience of a tertiary care hospital in Varanasi (India)

**DOI:** 10.1186/1471-230X-8-36

**Published:** 2008-08-20

**Authors:** Lekha Tuli, Anil K Gulati, Shyam Sundar, Tribhuban M Mohapatra

**Affiliations:** 1 Department of Microbiology, Institute of Medical Sciences, Banaras Hindu University, Varanasi – 221005, India; 2 Department of Medicine, Institute of Medical Sciences, Banaras Hindu University, Varanasi – 221005, India

## Abstract

**Background:**

Protozoan infections are the most serious among all the superimposed infections in HIV patients and claim a number of lives every year. The line of treatment being different for diverse parasites necessitates a definitive diagnosis of the etiological agents to avoid empirical treatment. Thus, the present study has been aimed to elucidate the associations between diarrhoea and CD4 counts and to study the effect of HAART along with management of diarrhoea in HIV positive patients. This study is the first of its kind in this area where an attempt was made to correlate seasonal variation and intestinal protozoan infestations.

**Methods:**

The study period was from January 2006 to October 2007 wherein stool samples were collected from 366 HIV positive patients with diarrhea attending the ART centre, inpatient department and ICTC of S.S. hospital, I.M.S., B.H.U., Varanasi. Simultaneously, CD4 counts were recorded to assess the status of HIV infection vis-à-vis parasitic infection. The identification of pathogens was done on the basis of direct microscopy and different staining techniques.

**Results:**

Of the 366 patients, 112 had acute and 254 had chronic diarrhea. The percentages of intestinal protozoa detected were 78.5% in acute and 50.7% in chronic cases respectively. Immune restoration was observed in 36.6% patients after treatment on the basis of clinical observation and CD4 counts. In 39.8% of HIV positive cases *Cryptosporidium *spp. was detected followed by *Microsporidia *spp. (26.7%). The highest incidence of intestinal infection was in the rainy season. However, infection with *Cyclospora *spp. was at its peak in the summer. Patients with chronic diarrhea had lower CD4 cell counts. The maximum parasitic isolation was in the patients whose CD4 cell counts were below 200 cells/μl.

**Conclusion:**

There was an inverse relation between the CD4 counts and duration of diarrhea. *Cryptosporidium *spp. was isolated maximum among all the parasites in the HIV patients. The highest incidence of infection was seen in the rainy season.

## Background

Since the beginning of the AIDS pandemic, opportunistic infections (O.I.'s) have been recognized as common complications of HIV infection. The spectrum of O.I.'s in the HIV infected subjects varies from one region to another [1]. Of these, opportunistic protozoan infections are the most serious ones causing severe morbidity and mortality. Reports indicate that diarrhoea occurs in 30–60% of patients in developed countries and in about 90% of AIDS patients in Haiti and Africa [2]. Given the similar background of poverty and malnutrition, the clinical picture of the disease in India parallels that of Africa.

There have been reports regarding frequency of various pathogens causing diarrhea from different parts of India [3-5]. However, there is paucity of data on correlations of CD4 levels and the etiology of diarrhea among the HIV patients of Eastern part of Uttar Pradesh, India. As most of the protozoan infections are treatable, it is important that an early and accurate diagnosis be made [6].

Thus, the present study was conducted to isolate and identify the protozoans causing diarrhoea in HIV patients so as to give an accurate diagnosis to avoid empirical treatment. An attempt was made to elucidate the associations between diarrhoea and CD4 counts and to study the effect of HAART as well as anti diarrhoeal treatments in these patients. For the first time an analysis of the seasonal variations in the occurrence of intestinal protozoan infections was also made as it holds epidemiological significance.

## Methods

This study was conducted from January 2006 to October 2007 in the Department of Microbiology and ART centre of S.S. Hospital, I.M.S., B.H.U., Varanasi, India. Being a tertiary care hospital, it caters to the patients from the neighbouring areas of U.P., M.P., Bihar, Jharkhand, Chattisgarh and Nepal. The study was a part of PhD work. For all assignments of this type institute ethical committee first review the protocol and approves it.

### Study cases

The stool samples were collected from 366 HIV positive patients attending the Anti Retroviral Therapy (ART) centre, inpatient department and the ICTC (Integrated Counselling and Testing Centre). All these patients came with the presenting complaint of diarrhoea and were investigated for the enteric protozoan as and when they reported. These patients were mostly promiscuous by habit.

The subjects who were HIV negative and without diarrhoea were not included in the study.

### Controls

Stool samples from 200 cases were collected from the non-HIV positive family members of the patients who had diarrhoea and were obviously from similar environmental, social and economic background.

A questionnaire was prepared to document the age, sex, route of transmission of infection and the demographic profile.

We also studied the effect of HAART and anti diarrhoeal preparations in these patients, as they were potential culprits for HAART and anti diarrhoeal treatment. All the patients on HAART were being treated with Ziduvudine, Lamivudine, Efavirenz and Nevirapine. The antiparasitic drugs prescribed were ornidazole, metrogyl, nitazoxamide and albendazole.

The patients were defined HIV seropositive if they tested positive for HIV infection by ELISA test (Micro Lisa – HIV, New Delhi, India) and a rapid test (HIV-Comb, New Delhi) [7].

### CD4 cell estimation

The CD4 cell count estimations were done by FACS Count (Becton Dickinson, Singapore). Each time the patients provided their stool samples, their most recent CD4 count was recorded for analysis. The CD4 cell counts were taken post therapy also in order to assess the immune restoration for case management.

The duration and episodes of recurrent diarrhoea were recorded thus classifying it as acute if it lasted for less than a month and chronic if it lasted for more than a month.

### Parasitological Examinations

Stool samples were collected in wide-mouthed disposable containers and processed immediately. If there was delay in processing the samples, they were preserved at 4°C. The consistency of the stool samples was recorded. A small portion of the sample was emulsified in a drop of saline and lugol's iodine on the slide and observed under the microscope. The samples were concentrated by Modified formol ether technique [8]. Thereafter, the samples were stained by modified acid fast and modified safranin technique [8]. The smears were microscopically examined. Screening for *Microsporidia *spores was done with the help of Calcoflour White staining method and identified on the basis of their size [8].

During parasitological analysis we correlated the occurrence of the parasites with the route of HIV transmission in the patients. The study was divided into three parts depending on the seasons viz. summer, rainfall and winter to correlate and study the possible role of seasonal variation in protozoan diarrhoea.

## Results

In the study we observed that there was a male preponderance 271/366, (74.0%) in the age group 31–40 years. Maximum patients (95%) belonged to Varanasi and the rural areas of Eastern UP.

A total of 366 patients were screened and of these 134(36.6%) patients, showed immune restoration, which clinically indicated positive response to HAART.

**Figure 1:**
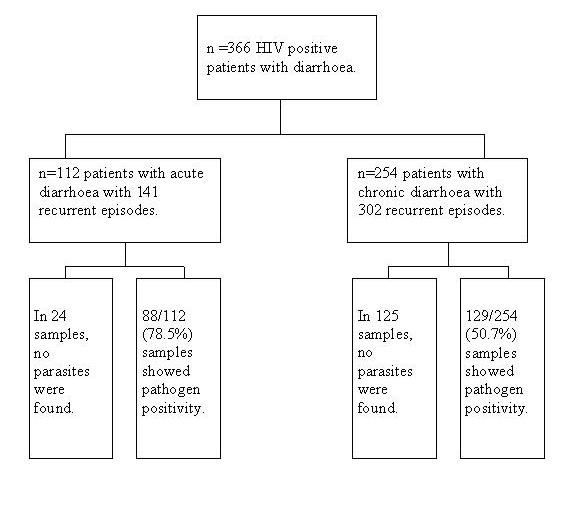
Flowchart showing the symptoms, diarrhoeal episodes and percentage of pathogen positivity among 366 HIV positive patients.

Of the 366 HIV positive patients with diarrhoea, 112 patients had episodes of acute and 254 had chronic diarrhoea. The details are given in a flowchart (Figure 1).

The macroscopic examination of the stool samples revealed that positivity of finding a pathogen in the sample was four times more in the case of watery samples as compared to the semi formed samples. *Cryptosporidium *spp. (39.8%) was the most commonly found parasite in the HIV positive patients followed by *Microsporidia *spp. (26.7%). There were (25.1%) cases of mixed infections. Of the mixed infections 7.65% cases showed presence of helminths like Hookworm, *H. nana *and *Trichuris trichiura *along with the enteric coccidian. The remaining 17.45% were mixed infections of *Cryptosporidium *spp., *Cyclospora *spp. and *Microsporidia *spp. The samples taken from the controls showed a predominance of helminthic infestation with *Ascaris lumbricoides *(22.0%) leading the list followed by Hookworm (20%). The data is shown in the ensuing table. (Table 1).

**Table 1:** Comparison of the parasites isolated from the stool samples of AIDS patients and normal controls.

Parasites isolated	Percentage of parasites isolated in HIV positive patients with diarrhoea. (n = 366)	Percentage of parasites isolated in HIV negative persons with diarrhoea. (n = 200)
*Cryptosporidium *spp.	146(39.8%)	42(21.0%)
*Microsporidia *spp.	98(26.7%)	--
*Cyclospora *spp.	88(24.0%)	3(1.3%)
*Giardia *spp.	37(10.1%)	--
*Entamoeba *spp.	11(3.0%)	4(2.0%)
*Isospora belli*	2(0.5%)	--
Hookworm	17(4.6%)	40(20.0%)
*Trichuris trichiura*	9(2.4%)	--
*Hymenolepsis nana*	2(0.5%)	6(3%)
*Ascaris lumbricoides*	--	44(22.0%)
Mixed infections	92(25.1%)	--

Figures in parentheses express the percentage (%) of parasites isolated.

The study revealed that Giardiasis was more commonly found in cases that had a history of homosexual practice. Of all the cases examined, 25/366 (6.8%) had acquired the infection through homosexual route and 315/366 (86.0%) through heterosexual route. Sixteen out of 25 (64%) homosexual men had diarrhea due to *Giardia *spp.

### Correlation between CD4 count, type and duration of diarrhoea

The CD4 levels were inversely proportional to the duration of diarrhoea. Patients with chronic diarrhoea had lower CD4 counts than those who had acute diarrhoea. Table 2 shows the association between types of diarrhoea, parasites isolated and CD4 counts of 366 AIDS patients.

**Table 2:** The associations between type of diarrhoea, parasites isolated and CD4 counts of 366 AIDS patients.

Parasites isolated	CD4 cells < 200 cells/μl		CD4 cells 200–350 cells/μl		CD4 cells 350–500 cells/μl		Total
	Acute cases	Chronic cases	Acute cases	Chronic cases	Acute cases	Chronic cases	
	n = 57	n = 179	n = 34	n = 42	n = 21	n = 33	

*Cryptosporidium *spp.	38/57 (66.6%)	56/179 (31.2%)	11/34 (32.3%)	20/42 (47.6%)	8/21 (38%)	13/33 (39.3%)	146
*Microsporidia *spp.	15/57 (26.3%)	68/179 (37.9%)	5/34 (14.7%)	7/42 (16.6%)	3/21 (14.2%)	--	98
*Cyclospora *spp.	4/57 (7.0%)	43/179 (24.0%)	3/34 (8.8%)	28/42 (66.6%)	5/21 (23.8%)	5/33 (15.5%)	88
*Giardia *spp.	11/57 (19.3%)	7/179 (3.91%)	8/34 (23.5%)	4/42 (9.5%)	4/21 (19.5%)	3/33 (9.5%)	37
*Entamoeba *spp.	4/57 (7%)	1/179 (0.5%)	5/34 (14.7%)	--	1/21 (4.7%)	--	11
*Isospora belli*	--	2/179 (1.1%)	--	--	--	--	2
Hookworm	1/57 (1.7%)	6/179 (3.3%)	--	5/42 (11.9%)	2/21 (9.5%)	3/33 (9%)	17
*Trichuris trichiura*	--	3/179 (1.6%)	4/34 (11.7%)	--	2/21 (9.5%)	--	9
*H. nana*	--	1/179 (0.5%)	--	1/42 (2.3%)	--	--	2

Total	73	187	36	65	25	24	410

Figures in parentheses express the percentage (%) of parasites isolated.

The incidence of diarrhea showed a distinct seasonality. Diarrhoea caused by *Cyclospora *spp. was found to be associated with increasing ambient temperatures. Of all the *Cyclospora *spp. isolated 63.6% were obtained from the patients in the summer season. A direct correlation was found between increased rainfall and isolation of *Cryptosporidium *oocysts (76.0%) and *Giardia *cysts (56.7%) in the stool samples collected from the patients having diarrhoea. However, no seasonal variation was observed in the occurrence of diarrhoea caused by *Microsporidia *spp. and other parasites. (Table 3).

**Table 3:** Correlation between seasonal variation and parasitic diarrhoea

Parasites	Summer		Rainfall		Winter	
Isolated	(March–June)		(July–October)		(Nov–Feb)	
	
	Cases	Controls	Cases	Controls	Cases	Controls
*Cryptosporidium *spp.	19 (5.1%)	13 (6.5%)	111 (30.3%)	23 (11.5%)	16 (4.3%)	6 (3%)
*Microsporidia *spp.	32 (8.7%)	--	38 (10.3%)	--	28 (7.6%)	--
*Cyclospora *spp.	56 (15.3%)	2 (1%)	25 (6.8%)	1 (0.5%)	7 (1.1%)	--
*Giardia *spp.	9(2.4%)	--	21(5.7%)	--	7(1.1%)	--
*Entamoeba *spp.	5(1.3%)	1(0.5%)	4(1.0%)	3(1.5%)	2(0.5%)	
*Isospora belli*	2(0.5%)	--	--	--	--	--
Hookworm	5 (1.3%)	10 (5%)	12 (3.2%)	27 (13.5%)	--	s3 (1.5%)
*Trichuris trichiura*	4(1.0%)	--	3(0.8%)	--	2(0.5%)	--
*H. nana*	--	--	2(0.5%)	4(2%)	--	2(1%)

Figures in parentheses express the percentage (%) of parasites isolated.

## Discussion

With the emergence of AIDS, parasitic diarrhea has gained significance, as it is one of the important causes of morbidity and mortality. The line of treatment being different for diverse parasites necessitates a definitive diagnosis and study of the etiological agents causing diarrhea, especially when it can be fatal in this vulnerable group of individuals.

Our study showed prevalence of more males than females (p < 0.05). Predominance of male cases may be due to their migration to the metropolitan cities in search of work. Staying away from the families for longer periods and males being promiscuous by habit resulted in them, acquiring HIV infection.

The drugs of choice for diarrhoea for practising clinician were ornidazole, metrogyl, nitazoxamide and albendazole. However, Sengupta et al observed that paramomycin was effective against cryptosporidiosis [9]. However, HAART added to the efficacy of the aforementioned anti protozoals and 36.6% patients were found to have a rebound in their CD4 cell counts. A study conducted by Guadalupe et al, showed that viral suppression is more effective in GALT (gut-associated lymphoid tissue) of patients with primary HIV infection than patients having chronic HIV infection during HAART [10]. They found delay in restoration of gut mucosal immune system of patients with chronic infection as gut acts as a viral reservoir and keeps from eradicating the virus.

In this study we came across patients ranging from initial to advanced stages of the disease. There were 69.3% patients with chronic and 30.6% patients with acute diarrhea (p < 0.05). Recurrent episodes and presence of diarrhea even at higher CD4 levels can be attributed to reduced intestinal mucosal immunity [11].

The percentage of parasite isolation in our study was 78.5% in acute and 50.7% in chronic cases. A similar study conducted in the same set up by Attili et al showed lower isolation rates [12]. This discordance in the results could be due to more than one technique used in this study for identification, which might have increased the sensitivity. Lower isolation rate of parasites in chronic cases was because most of the patients with chronic diarrhoea were on empirical antidiarrheal treatment. Moreover, in this country anti-diarrhoeal drugs are freely available across the counter in the drug store even without prescription.

In this study it was observed that probability of finding a pathogen from watery and semi formed stools was four times greater as compared to formed stools [12]. This can be attributed to greater shedding, more inflammatory response and greater virulence of the pathogens causing watery diarrhea.

Samples collected from the controls coming from the same environmental background helped in tracing the source of infection. Parasite like *Cryptosporidium *spp. isolated from both the groups indicated water as the main source of infection, which highlighted poor sewage disposal practices and sewage spills. Presence of Hookworm indicated lack of sanitation and low socio-economic status of the cases coming from rural areas.

*Cryptosporidium *spp. (39.8%) was the most commonly isolated protozoan followed by *Microsporidia *spp. (26.7%). As compared to the controls, the observed incidence of these organisms in HIV patients was significantly higher (p < 0.05). Another study conducted by Samantaray et al, also showed similar isolation rates in HIV patients [13] whereas, in a study of Southern India lesser number of *Cryptosporidium *spp. (9%) were isolated [14]. A study conducted in Mumbai showed the infection rate of *Microsporidia *spp. in HIV patients as 17.18% [15]. On the contrary our study detected 26.7% of *Microsporidia *spp. This increase in isolation rates could be due to the fact that the numbers of cases studied were much higher in our study as compared to the study of Siddhartha et al [15]. When compared to other studies of Southern India, isolation rate of *Isospora belli *(0.5%) was lower in our study [14]. This discrepancy in the findings maybe attributed to geographical variation. Calcoflour White staining technique, which is a screening method, identified the Microsporidia spp. However, its presence will be confirmed later by Chromotrope 2R staining method in order to avoid any false positive results, if any. The clinical picture and the microscopic examination of the dysenteric stools revealed haematophagous trophozoites suggesting that the *Entamoeba *spp. isolated were presumably that of *Entamoeba histolytica*. This screening method was adopted due to lack of facilities for isoenzyme analysis and other tests to differentiate it from *Entamoeba dispar *[16]. Although the study was conducted to screen for the enteric protozoan, we came across 7.65% cases where helminths like Hookworm, *H. nana *and *Trichuris trichiura *co-existed with protozoa. These were probably flushed from the intestine because of diarrhoea or expelled after treatment. Therefore, we reported their presence as and when we came across the helminths during stool examination.

It was found that 6.8% patients had acquired the HIV through homosexual route. *Giardia *spp. (64%) was present more in this group of people as compared to those who had heterosexual practice. Our findings are in accordance with Curry et al [6].

The maximum parasitic isolation was in the group of patients who had CD4 cell counts below 200 cells/μl and *Cryptosporidium *spp. was found to be the most commonly acquired protozoa causing chronic diarrhoea. The isolation rates decreased with the increase in the CD4 cell counts. This finding is in accordance with the study conducted by Attili et al. They also found an inverse correlation between CD4 counts and isolation rates of parasites from diarrhoea patients [12].

Key climatic variables, particularly humidity and temperatures have always had a relationship to waterborne diseases. The Milwaukee episode of 1993, which affected 403,000 people, is a commonly quoted waterborne Cryptosporidiosis outbreak [17]. The advent of rainy season marks the beginning of many infectious diseases. In our work too, the maximum parasitic isolation was during rainfall, with *Cryptosporidium *spp. at the top of the list followed by *Giardia *spp. (p < 0.05). We observed that isolation rate of *Cyclospora *spp. peaked during the summer and was significantly higher as compared to in the other seasons (p < 0.05). This is because sporulation or maturation of the immature oocysts excreted in the faeces depends on warm temperatures [17].

### Limitations of the study

1. Immune restoration as detected by CD4 counts was clinically assessed.

2. Calcoflour White staining for *Microsporidia *spp. is merely a screening method. However, the authors have plans to carry out identification techniques like Chromotrope 2R staining to confirm the results.

3. The study was done as and when the symptoms of diarrhoea appeared and accordingly they were categorised season wise. It is difficult to establish the time period of initiation of infection.

## Conclusion

From this study it appears that the CD4 counts and prevalence of the protozoal infection in a particular geographic area should be considered before instituting empirical therapy to the AIDS patients attending the ART centre.

## Competing interests

The authors declare that they have no competing interests.

## Authors' contributions

All the authors read and approved the final manuscript. LT designed the study, performed the experimental work, conceived and drafted the manuscript. AKG provided the CD4 cell count data and helped to edit the manuscript, SS participated in coordination of the study and provided the clinical data and TMM supervised the study design, coordination of the study and helped to edit the manuscript.

## Pre-publication history

The pre-publication history for this paper can be accessed here:



## References

[B1] Vajpayee N, Kanswal S, Seth P, Wig N (2003). Spectrum of Opportunistic Infections and Profile of CD4^+ ^counts among AIDS patients in North India. Infection.

[B2] Framm SR, Soave R (1997). Agents of diarrhea. Med Clin North Am.

[B3] Joshi M, Chowdhary AS, Dalar PJ, Maniar JK (2002). Prevalence of intestinal parasitic pathogens in HIV-seropositive individuals in Northern India. Natl Med J India.

[B4] Lanjewar DN, Rodrigues C, Saple DG, Hira SK, Dupont HL (1996). Cryptosporidium, Isospora and Strongyloides in AIDS. Natl Med J India.

[B5] Mohandas, Sehgal R, Sud A, Malla N (2002). Prevalence of intestinal parasitic pathogens in HIV-seropositive individuals in northern India. Jpn J Infect Dis.

[B6] Curry A, Turner AJ, Lucas S (1991). Opportunistic protozoan infections in human immunodeficiency virus disease: Review highlighting diagnostic and therapeutic agents. J Clin Pathol.

[B7] Government of India (1999). Epidemiology of HIV/AIDS.p 1–11 In B.B. Rewari(ed), Specialists Training and Reference Module.

[B8] Diagnostic Procedures for Stool Specimens. http://www.dpd.cdc.gov.

[B9] Sengupta D, Lal S, Shrinivas (2002). Opportunistic Infections in AIDS. Natl Med J India.

[B10] Guadalupe M, Sankaran S, George MD, Reay E, Verhoeven D, Shacklett BL, Flamm J, Wegelin J, Prindiville T, Dandekar S (2006). Viral Suppression and Immune Restoration in the Gastrointestinal Mucosa of Human Immunodeficiency Virus Type 1-Infected Patients Initiating Therapy during Primary or Chronic Infection. Journal of Virology.

[B11] Schneider T, Jahn H, Schmidt W, Riecken E, Zeitz M, Ullrich R (1995). Loss of CD4 cells in patients infected with HIV is more pronounced in mucosa than in blood. GUT.

[B12] Satya V Suresh Attili, Gulati AK, Singh VP, Varma DV, Rai M, Sundar S (2006). Diarrhoea, CD4 counts and enteric infections in a hospital based cohort of HIV-infected patients around Varanasi, India. BMC Infectious Diseases.

[B13] Samantaray JC, Panda PL (2005). Spectrum of Intestinal Parasitosis in Immunocompromised Patients Suffering from Diarrhea: with special reference to Cryptosporidium, Isospora, Cyclospora, Microsporidia and Blastocystis. Manuals on Indo-US Workshop on Diarrhea and Enteric Protozoan Parasites: Indian Council of Medical Research.

[B14] Ballal M (2005). Opportunistic intestinal protozoal infections in HIV infected patients in a rural cohort population in Manipal, Karnataka-Southern India. Manuals on Indo-US Workshop on Diarrhea and Enteric Protozoan Parasites. Indian Council of Medical Research.

[B15] Dalvi S, Mehta P, Koticha A, Gita N (2006). Microsporidia as An Emerging Cause of Parasitic Diarrhoea in HIV Seropositive Individuals in Mumbai. Bombay Hospital journal.

[B16] Bansal D, Sehgal R, Chawla Y, Mahajan RC, Malla N (2004). In vitro activity of antiamoebic drugs against clinical isolates of *Entamoeba histolytica *and *Entamoeba dispar*. Annals of Clinical Microbiology and Antimicrobials.

[B17] Rose JB, Epstein PR, Lipp EK, Sherman BH, Bernard SM, Patz JA (2001). Climatic Variability and Change in the United States: Potential Impacts on Water- and Foodborne Diseases Caused by Microbiologic Agents. Environ Health Perspect.

